# Identification of a Role for the Ventral Hippocampus in Neuropeptide S-Elicited Anxiolysis

**DOI:** 10.1371/journal.pone.0060219

**Published:** 2013-03-28

**Authors:** Julien Dine, Irina A. Ionescu, Jens Stepan, Yi-Chun Yen, Florian Holsboer, Rainer Landgraf, Matthias Eder, Ulrike Schmidt

**Affiliations:** 1 RG Neuronal Network Dynamics, Max Planck Institute of Psychiatry, Munich, Germany; 2 RG Molecular Psychotraumatology, Max Planck Institute of Psychiatry, Munich, Germany; 3 RG Neuronal Plasticity, Max Planck Institute of Psychiatry, Munich, Germany; 4 Max Planck Institute of Psychiatry, Munich, Germany; 5 RG Behavioral Neuroendocrinology, Max Planck Institute of Psychiatry, Munich, Germany; Nathan Kline Institute for Psychiatric Research and New York School of Medicine, United States of America

## Abstract

Neuropeptide S (NPS) increasingly emerges as a potential novel treatment option for anxiety diseases like panic and posttraumatic stress disorder. However, the neural underpinnings of its anxiolytic action are still not clearly understood. Recently, we reported that neurons of the ventral hippocampus (VH) take up intranasally administered fluorophore-conjugated NPS and, moreover, that application of NPS to mouse brain slices affects neurotransmission and plasticity at hippocampal CA3-CA1 synapses. Although these previous findings define the VH as a novel NPS target structure, they leave open whether this brain region is directly involved in NPS-mediated anxiolysis and how NPS impacts on neuronal activity propagation in the VH. Here, we fill this knowledge gap by demonstrating, first, that microinjections of NPS into the ventral CA1 region are sufficient to reduce anxiety-like behavior of C57BL/6N mice and, second, that NPS, via the NPS receptor, rapidly weakens evoked neuronal activity flow from the dentate gyrus to area CA1 *in vitro*. Additionally, we show that intranasally applied NPS alters neurotransmission and plasticity at CA3-CA1 synapses in the same way as NPS administered to hippocampal slices. Thus, our study provides, for the first time, strong experimental evidence for a direct involvement of the VH in NPS-induced anxiolysis and furthermore presents a novel mechanism of NPS action.

## Introduction

Neuropeptide systems receive increasing attention as potential novel pharmacotherapeutic options for the treatment of pathological anxiety, which is a core symptom of anxiety diseases like panic and posttraumatic stress disorder (PTSD) [1], [2]. In this context, the neuropeptide S (NPS) system is of special interest as it has been well established that NPS exerts pronounced anxiolytic effects in rodents both after central [3], [4] and after intranasal administration [5], [6]. However, the mechanisms underlying NPS-elicited anxiolytic effects still remain elusive. We recently demonstrated that intranasally applied fluorescent Cy3-NPS is taken up into neurons not only in various mouse brain regions previously known as NPS receptor (NPSR) expression sites like the amygdala and the hypothalamic nuclei [7], [8], but interestingly also in the ventral hippocampus (VH) [5]. The VH, which has been described as a player in the anxiety network both in rodents and humans [9]–[11], has not been associated with NPS actions to date, although this limbic region is well known as a target of other neuropeptides like oxytocin [12], arginine vasopressin [13], NPY and cholecystokinin (CCK) [14]. Interestingly, CCK8 microinfusions into the VH were reported to induce anxiety-like behavior in rats [15]. However, reports on the mechanisms underlying VH-mediated anxiolysis are scarce [9], whereas the role of the amygdala and the prefrontal cortex (PFC) in anxiety regulation and anxiety disorders has been widely studied and confirmed [16]–[19]. In addition to identifying NPS target neurons in the VH, we lately demonstrated that bath application of NPS to mouse brain slices decreases paired-pulse facilitation and long-term potentiation (LTP) at CA3-CA1 synapses. These NPS effects were inhibited by the specific NPSR antagonist (R)-SHA 68 [5]. Analyses of NPSR knockout mice revealed NPSR to be the sole mediator of NPS effects [20], [21]. Our previous findings leave the following questions open: First, whether intranasally applied NPS leads to the same effects on neurotransmission and plasticity at CA3-CA1 synapses in awake mice as NPS bath application to murine hippocampal slices; second, whether the VH is directly involved in NPS-mediated anxiolysis; and third, how NPS influences neuronal activity flow within the hippocampus. Here, we fill these knowledge gaps, first, by analyzing potential changes in input-output relationships, paired-pulse facilitation and LTP at CA3-CA1 synapses in brain slices prepared from C57BL/6N mice previously treated intranasally with NPS; second, by testing whether microinjections of NPS directly into the ventral CA1 region (vCA1) are sufficient to reduce anxiety-like behavior in C57BL/6N mice mice; and third, by investigating whether and how NPS influences evoked neuronal activity propagations from the dentate gyrus (DG) to the CA1 subfield.

## Materials and Methods

### Animals

All experiments were performed in adult (10- to 12-week-old) male mice. For behavioral experiments, C57BL/6N mice were purchased from Charles River Germany GmbH (Sulzfeld, Germany). For all other experiments, we used C57BL/6N animals bred in the animal facility of the Max Planck Institute of Biochemistry (Martinsried, Germany). All mice were housed individually for at least 6 days before the start of the experiments, on a 12 h light/dark cycle with food and water *ad libitum*. All procedures were approved by the Government of Upper Bavaria and were in accordance with European Union Directive 86/609/EEC.

### Chemicals

Cy3-NPS was purchased from Phoenix Pharmaceuticals (Karlsruhe, Germany) and rat NPS from Bachem (Bubendorf, Switzerland). Both were dissolved in artificial cerebrospinal fluid (ACSF, for composition see below) to the desired final concentration. DAPI, Di-4-ANEPPS and all salts for the ACSF were purchased from Sigma-Aldrich (Taufkirchen, Germany). A 20.8 mM stock solution of Di-4-ANEPPS was prepared in dimethylsulfoxide (DMSO). The active enantiomer of the specific NPSR antagonist SHA 68, (R)-SHA 68 [22], [23], was a generous gift from A. Sailer (Novartis, Basel, Switzerland). (R)-SHA 68 was dissolved in DMSO and diluted for use in ACSF at a final concentration of 10 µM (<0.1% DMSO).

### Surgery

Surgery was performed as previously described [5]. Briefly, 23 gauge guide cannulas were implanted in the CA1 region of the VH at the following coordinates: 3.1 mm posterior and ±3 mm lateral from bregma, and 2 mm ventral from the skull surface [24]. For behavioral experiments, animals were implanted bilaterally; for Cy3-NPS injections, implantation was performed unilaterally. The animals were allowed to recover for at least 6 days before the behavioral experiments.

### Administration of Cy3-NPS and brain section processing

Cy3-NPS was administered unilaterally at a concentration of 0.07 nmol in a volume of 0.7 µl ACSF at the following coordinates: 3.1 mm posterior and ±3 mm lateral from bregma, and 4.5 mm ventral from the skull surface [24]. Mice were sacrificed 30 min after application. Brains were removed and post-fixed in 4% paraformaldehyde overnight at 4°C, then shock-frozen in methylbutane and stored at −80°C. 40 µm cryosections were obtained and processed as previously described [5]. Images were acquired with a confocal microscope (Olympus IX81, software: FluoView FV1000 2.1.2.5).

### Behavioral experiments

Mice were injected bilaterally either with 0.1 nmol NPS in 0.5 µl ACSF per side or with 0.5 µl of ACSF per side at the same coordinates as used for the Cy3-NPS injections (see above). 30 min after injection, three behavioral assays (open field, dark-light test, and elevated plus-maze (EPM)) were performed sequentially in the order mentioned. Each test lasted 5 min, with a 5 min break in between, as described previously [5]. Animal behavior was videotaped and relevant parameters were analyzed using the tracking software ANY-maze version 4.30 (Stoelting, Wood Dale, IL, USA) [25]. Mice were sacrificed 24 h after completion of the behavioral assays and the locations of the guide cannulas and injection sites were checked in histological cryosections of 40 µm counterstained with DAPI. Mice with deviating injection sites were excluded from further analysis.

### Intranasal administration of NPS

was performed as previously described [5]. Briefly, alert mice, habituated to handling, were restrained manually during the administration procedure in a supine position with the head immobile at an angle of approximately 45° to the body (Figure S1). 7 µl of NPS (7 nmol) or ACSF alone were pipetted alternatingly to each nostril without touching the nasal mucosa; after 5 min, the procedure was repeated. Mice were then allowed to rest for 2 h before slice preparation.

### Electrophysiology

Field excitatory postsynaptic potential (fEPSP) recordings in horizontal brain slices (350 µm-thick) containing the VH were performed as previously described [26]. Only the first two slices from the ventral surface of the brain in which the CA1 region was clearly visible were used for the measurements. Hippocampal slices were continuously perfused (4–5 ml/min flow rate) at room temperature (23–25°C) in a submerged chamber with carbogenated (95% O_2_/5% CO_2_) ACSF containing (in mM): NaCl, 125; KCl, 2.5; NaH_2_PO_4_, 1.25; NaHCO_3_, 25; MgCl_2_, 1; CaCl_2_, 2; and glucose, 25 (pH 7.4). Square pulse electrical stimuli (0.066 Hz, 50 µs) were delivered to the stratum radiatum of the CA1 subfield and evoked fEPSPs were recorded. In all experiments (to allow comparison between the NPS- and vehicle-treated animals) the stimulation intensity was set to the half maximum intensity at which population spikes appeared. The paired-pulse ratio was calculated as fEPSP2 amplitude/fEPSP1 amplitude. LTP was induced by high-frequency stimulation (HFS, 100 stimuli at 100 Hz).

### Voltage-sensitive dye imaging (VSDI)

According to Maggio and Segal [27] and Fanselow and Dong [28], VSDI experiments were conducted in the VH. Horizontal brain slices (350 µm-thick) were prepared as previously described [29]–[31]. For staining, slices were kept for 15 min in carbogenated ACSF containing the voltage-sensitive dye Di-4-ANEPPS (7.5 µg/ml, <0.1% DMSO), before being stored for at least 30 min in normal ACSF. VSDI and data analysis were performed using the MiCAM02 hard- and software package (BrainVision, Tokyo, Japan). The tandem-lens fluorescence microscope was equipped with the MiCAM02-HR camera and the 2× and 1× lens at the objective and condensing side, respectively (for further technical details see http://www.scimedia.com). Acquisition settings were: 88×60 pixels frame size, 36.4×40.0 µm pixel size, and 2.2 ms sampling time. To reduce noise, four acquisitions subsequently recorded at intervals of 5 s were averaged. Neuronal activity was evoked by square pulse electrical stimuli (200 µs, 15–20 V) delivered to the DG granule cell layer via a custom-made monopolar tungsten electrode (Teflon-insulated to the tip of 50 µm diameter). From recorded signals, the fractional change in fluorescence (Δ*F*/*F*) was calculated. For all quantifications, Δ*F*/*F* values were spatially and temporally smoothed using a 3×3×3 average filter. VSDI signals presented in images were smoothed with a 5×5×3 average filter. Pixelation of images was reduced by the interpolation function of the MiCAM02 software. For analysis of neuronal population activity in hippocampal subregions, three standardized circular regions of interest (ROIs) were manually set according to anatomical landmarks. The first ROI (*r* = 3 pixels), termed ‘Hilus’, was placed centrally into the hilus of the DG, between the tip of the stimulation electrode and the proximal end of the CA stratum pyramidale. The second ROI ‘CA3’ (*r* = 6 pixels) was positioned into the CA3 region near the DG, but not overlapping with it. The third ROI ‘CA1’ (*r* = 6 pixels) was placed into the CA1 subfield with a distance of approximately 400 µm from the visually identified distal end of the CA3 region. Both the ‘CA3’ and the ‘CA1’ ROI spanned the stratum oriens, stratum pyramidale, and stratum radiatum (lucidum). The average of smoothed Δ*F*/*F* values within a particular ROI served as final measure of neuronal population activity.

### Statistics

Statistical analysis was performed using Sigma Stat 3.5 and GraphPad Prism 5.03. Statistical significance was assessed by means of the two-tailed unpaired Student's t-test, except for the VSDI experiments for which the two-tailed paired Student's t-test was used. Data are given as mean ± SEM. In all graphs, *p* values are depicted as follows: *p<0.05, **p<0.01, ***p<0.001.

## Results

### Intranasally applied NPS impacts on basal glutamatergic neurotransmission and short- and long-term plasticity at CA3-CA1 synapses of the VH

First, to corroborate our previous findings on the role of the VH in the effects of intranasally administered NPS, we analyzed whether intranasally applied NPS, like bath-applied NPS (1 µM) [5], also impacts on paired-pulse facilitation and LTP at CA3-CA1 synapses. To this end, we performed field potential recordings in VH slices obtained from C57BL/6N mice which had been previously treated intranasally with 14 nmol NPS or vehicle. We decided to conduct the electrophysiological measurements 4 h after treatment since we determined earlier that intranasally applied NPS leads to a statistically significant anxiolytic effect 4 h after application [5]. In our previous study, we demonstrate that the specific NPSR antagonist (R)-SHA 68 prevents NPS-mediated modulation of basal glutamatergic neurotransmission and plasticity.

**Figure 1:**
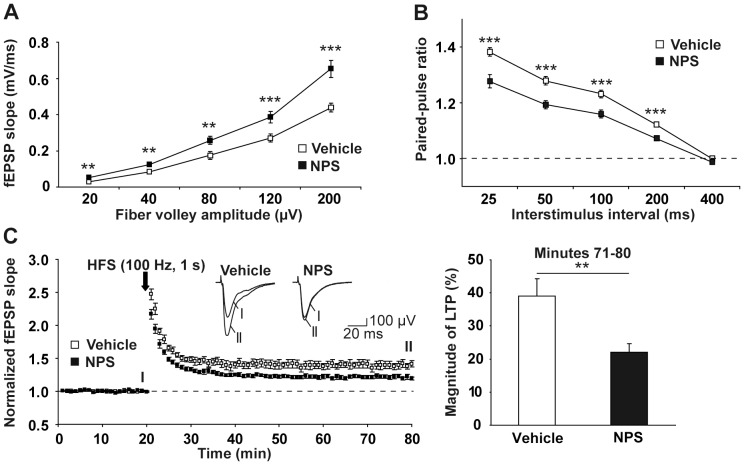
Intranasally applied NPS impacts on basal glutamatergic neurotransmission and plasticity at CA3-CA1 synapses of the VH in C57BL/6N mice. A, Intranasal NPS administration caused a shift of the input-output curve towards bigger fEPSP amplitudes (Vehicle: open squares: n = 12 slices from 7 mice; NPS: closed squares: n = 11 slices from 5 mice). B, Intranasally applied NPS reduced paired-pulse facilitation at interstimulus intervals of 25, 50, 100, and 200 ms (Vehicle: open squares: n = 14 slices from 7 mice; NPS: closed squares: n = 11 slices from 5 mice). C, Intranasal NPS application decreased the magnitude of LTP at CA3-CA1 synapses induced by high-frequency stimulation (HFS) (Vehicle: open squares: n = 10 slices from 5 mice, LTP magnitude for minutes 71–80 = 39±5%; NPS: closed squares: n = 10 slices from 5 mice, LTP magnitude for minutes 71–80 = 22±3%; p = 0.009, t = 2.944, df = 18).

As shown in Figure 1, intranasal administration of NPS to C57BL/6N mice led to weakening of both paired-pulse facilitation and LTP at CA3-CA1 synapses (values for LTP: 39±5.23% for vehicle-treated *vs.* 22±2.62% for NPS-treated animals, *p* = 0.009). Analysis of input-output relationships at these synapses showed that intranasal NPS application induced a shift of input-output curves towards bigger fEPSP amplitudes (Figure 1). Taken together, both bath-applied [5] and intranasally applied NPS influence basal glutamatergic neurotransmission and plasticity at CA3-CA1 synapses in the same direction.

### Microinjections of NPS into the VH reduce anxiety-like behavior

**Figure 2:**
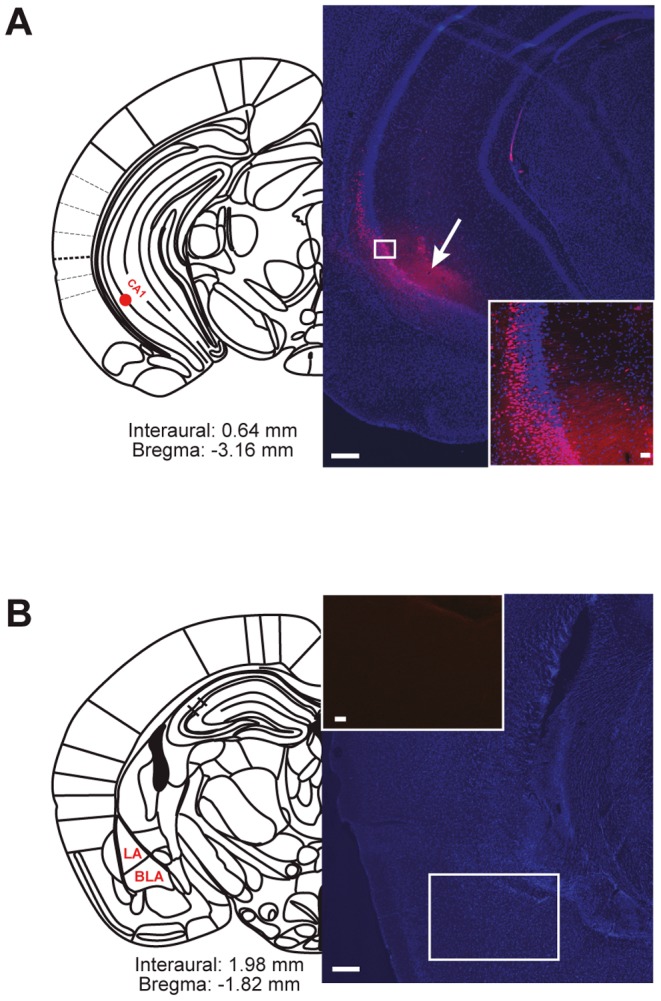
Cy3-NPS is locally restricted to the site of injection into area CA1 of the VH. A, Injection site on an anatomical plate [24]. Overlay of DAPI (nuclear staining, blue) and Cy3-NPS (red) signals. Arrow indicates the injection site in the brain section. B, Anatomical plate showing the lateral (LA) and basolateral (BLA) amygdala, and overview of the amygdala in a brain section after Cy3-NPS injection (inset: Cy3 channel only). n = 4. Scale bars, 200 and 20 µm.

Next, we wanted to find out whether the VH is directly involved in NPS-mediated anxiolysis. Before examining whether microinjections of NPS into the CA1 subfield of the VH modulate anxiety in C57BL/6N mice, we analyzed the distribution of the injected NPS using a fluorescent conjugate, i.e. Cy3-NPS. 30 min after injection, Cy3-NPS remained locally restricted to the VH and accumulated in single neurons of the hippocampal pyramidal, radiate, and oriens layers (red fluorescence in Figure 2A). After intrahippocampal injection, we never observed an uptake of Cy3-NPS in nuclei of the amygdala (Figure 2B).

**Figure 3:**
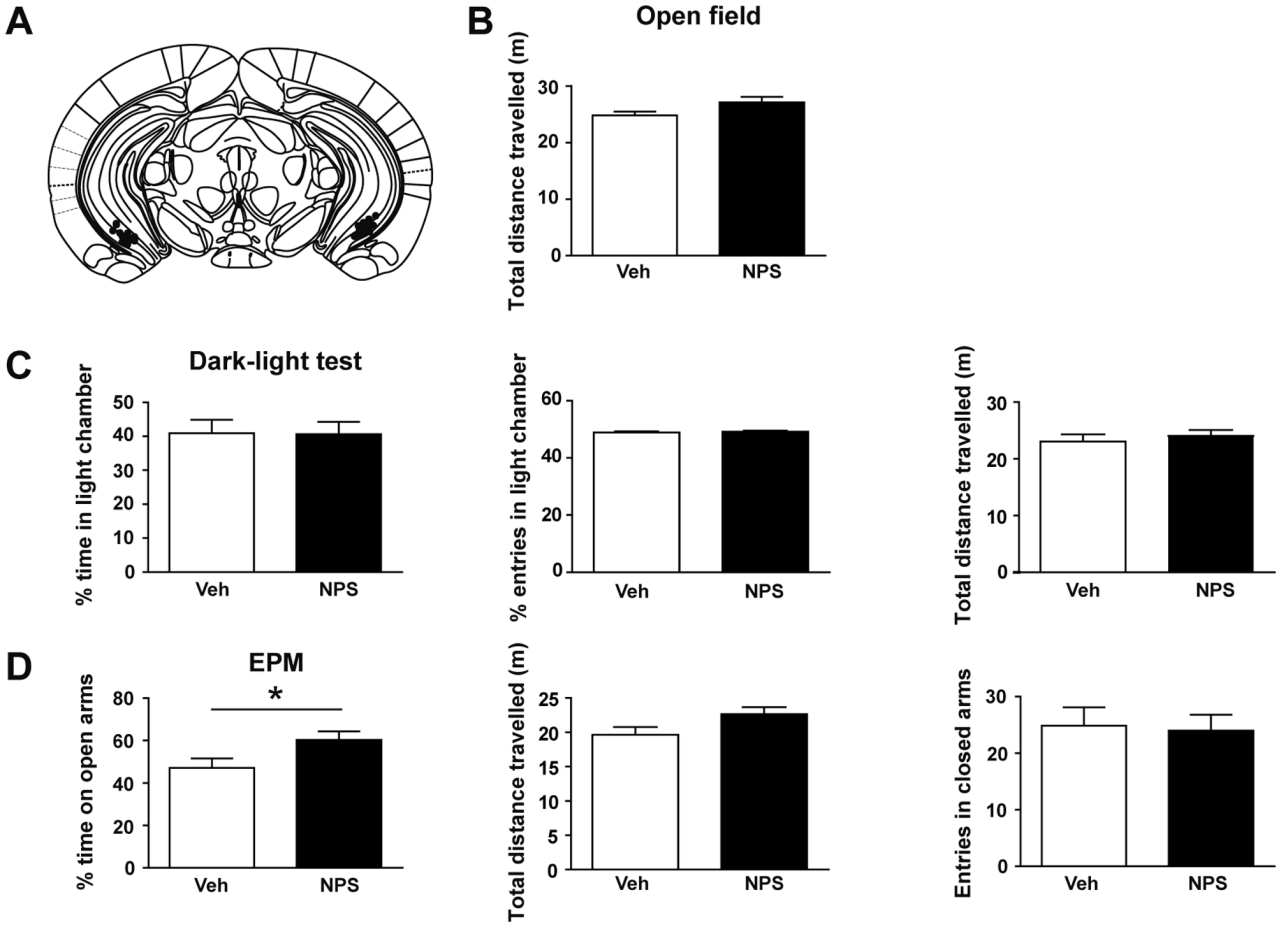
Microinjections of NPS into the VH reduce anxiety-like behavior in C57BL6/N mice. A, Anatomical plate showing the injection sites (n = 8 mice per group). B, The distance travelled in the open field was not changed by NPS injection (p = 0.074, t = 1.928, df = 14). C, Anxiety- and locomotion-related behavior in the dark-light test was not altered by NPS injection (parameters for anxiety-like behavior: % time in light chamber p = 0.964, t = 0.046, df = 14 and % entries in light chamber p = 0.553, t = 0.608, df = 14; parameter for locomotion: total distance travelled p = 0.54, t = 0.63, df = 14). D, NPS injections decreased anxiety-related behavior on the EPM (parameter for anxiety-like behavior: % time on open arms p = 0.042, t = 2.227, df = 14) without affecting locomotion (parameters for locomotion: total distance travelled p = 0.073, t = 1.94, df = 14 and entries in closed arms p = 0.842, t = 0.203, df = 14).

Thereupon, we investigated whether injections of unlabeled NPS into the vCA1 region produce similar anxiolytic effects as seen after intra-amygdalar [7] and intracerebroventricular (ICV) injections [3], [4], [32] as well as after intranasal administration [5], [6]. To this end, we employed standardized paradigms to study anxiety-related behavior 30 min after injection, as this time-point has been repeatedly shown to be optimal for measuring behavioral changes after intracerebral NPS injection [3], [7]. Anxiety- and locomotion-related parameters were examined in both the dark-light test and the EPM. Basal locomotion was determined by means of the open field test. Intra-vCA1-injected NPS did not significantly alter locomotion in any of the three tests (Figure 3B–D). However, most interestingly, NPS treatment elicited a significant anxiolytic effect on the EPM, as evident from an increase in the percentage of time spent on the open arms (Figure 3D, *p* = 0.042). These results are in accordance with our previous findings demonstrating that intranasally applied NPS causes the strongest anxiolytic effect on the EPM [5].

### NPS weakens neuronal activity flow from the DG to area CA1

Finally, we analyzed the influence of NPS on neuronal activity flow in the VH. Field potential recordings, as employed to generate the results shown in Figure 1, are a valuable tool to uncover changes in basal synaptic transmission and plasticity, but they are not suited to unveil alterations in neuronal network dynamics, which might be a closer neurophysiological correlate of behavior [29], [33], [34]. We recently established a high-speed voltage-sensitive dye imaging (VSDI) assay in mouse brain slices enabling the investigation of evoked neuronal activity propagations from the DG to area CA1 [29], [30], which is of high physiological relevance since the DG represents the major input region and area CA1 an important output subfield of the hippocampus [28]. We analyzed stimulus-evoked fast, depolarization-mediated imaging signals (‘FDSs’), which reflect neuronal action potentials and EPSPs [29]–[31], [33].

**Figure 4:**
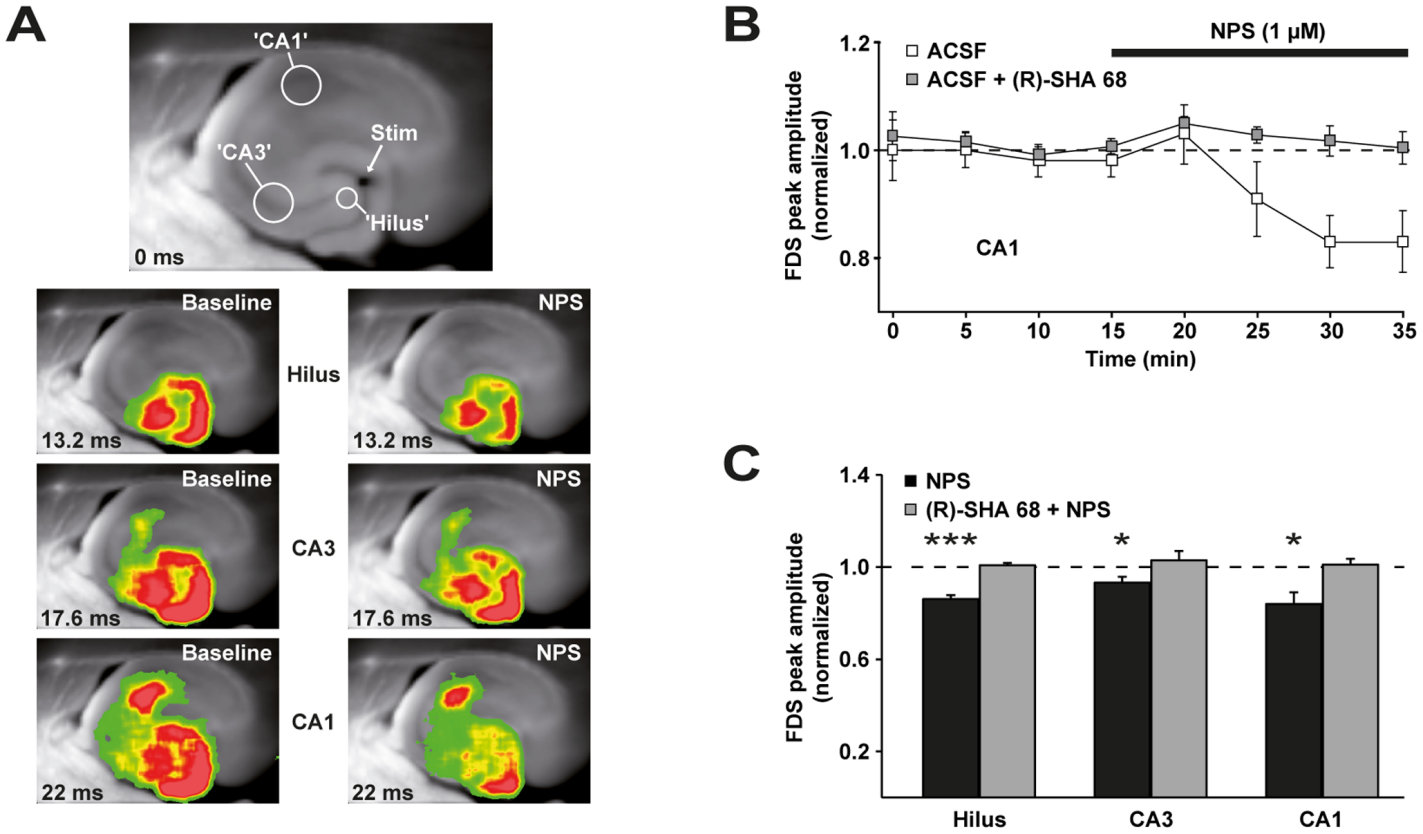
NPS rapidly weakens evoked neuronal activity flow from the DG to area CA1 in hippocampal slices prepared from C57BL/6N mice. A, (Upper panel) Illustration of the position of the stimulation electrode (Stim) and the three ROIs used for the calculation of neuronal population activity within the dentate hilus, the CA3 subfield, and area CA1. (Lower panels) Representative filmstrips depicting the propagation of VSDI signals from the DG to the CA1 region before (‘Baseline’) and after bath application of 1 µM NPS (‘NPS’). Warmer colors represent stronger neuronal activity. Time specifications are given relative to the electrical stimulation pulse. B, Time course of the experiments depicted for the CA1 output subfield of the VH. NPS (1 µM) decreased FDS peak amplitudes (84±5% of baseline, n = 7 slices from 6 mice). This effect was completely abolished by pretreatment (15 min) of slices with the specific NPSR antagonist (R)-SHA 68 (10 µM) (101±3% of baseline, n = 5 slices from 4 mice). Data were normalized to the mean FDS peak amplitude of the last two acquisitions during baseline recording. C, Quantification of NPS effects on FDS peak amplitudes in the dentate hilus, the CA3 region, and area CA1. NPS decreased FDS peak amplitudes in the dentate hilus to 86±2% (p<0.001, t = 8.357, df = 6), in CA3 to 93±3% (p = 0.037, t = 2.664, df = 6), and in CA1 to 84±5% (p = 0.019, t = 3.165, df = 6) of baseline values. These effects were completely abolished by the NPSR antagonist (R)-SHA 68 (10 µM). Statistical evaluation was performed by comparing the mean FDS peak amplitudes of the last two acquisitions during baseline recording with the mean FDS peak amplitudes of the last two acquisitions during application of NPS.

Bath application of NPS (1 µM) to VH slices rapidly weakened the stimulus-evoked activity flow from the DG to the CA1 subfield (Figure 4A, B). This effect was completely abolished by the specific NPSR antagonist (R)-SHA 68 (10 µM) (Figure 4B, C), proving that the effects observed are mediated by the NPS/NPSR complex. NPS reduced the amplitude of FDSs in the dentate hilus, the CA3 region, and area CA1, indicating that NPS effects on neuronal activity in the VH impact on the entire ventral trisynaptic circuit (Figure 4A, C).

## Discussion

Our findings show for the first time that intranasally applied NPS decreases short- and long-term plasticity at CA3-CA1 synapses in the VH, that microinjections of NPS restricted to the VH reduce anxiety-like behavior, and that NPS rapidly weakens neuronal activity flow from the DG to area CA1 in the VH.

This study uncovers a novel NPS mechanism of action by showing that NPS, via NPSR, induces a rapid decrease (within 5 min) in neuronal activity flow from the DG to the CA1 region of the VH (Figure 4). This observation is in line with our previous finding that intranasally and ICV applied Cy3-NPS accumulates *inter alia* in principal neurons of these hippocampal subfields [5]. Recently, using an identical approach, we demonstrated anxiogenic corticotropin-releasing factor (CRF) [35], [36] to enhance neuronal activity flow from the DG to the CA1 region [29], [30]. The opposite influences of anxiogenic CRF and anxiolytic NPS on activity propagation in the above mentioned hippocampal subfields warrant the speculation that modulation of neuronal activity flow in the VH might play a principal role in regulating anxiety-related behavior in rodents and, as the anatomy of the hippocampus is highly conserved across mammals [37], possibly also in humans. This speculation is supported by a study which, also employing a VSDI paradigm, demonstrated that alterations in the strength of evoked activity propagation within the DG relative to the hippocampal CA1 output subfield accompany stress-induced behavioral changes in a rat model of major depression [33], which, like anxiety diseases, also belongs to the group of affective disorders.

An NPS-responsive network, encompassing parts of the amygdala, such as the basolateral (BLA) and lateral (LA) nuclei, has already been identified by others [7], [38], [39] and intra-amygdalar injection of NPS was shown to be sufficient to elicit anxiolytic-like behavior in mice [7], [38]. The NPS-induced reduction of neuronal activity flow from the DG to area CA1 might possibly contribute to a decrease in the activity of amygdalar anxiety circuits, since CA1 pyramidal cells of the VH are known to form excitatory synapses with amygdalar neurons [28], [40].

Additionally, we observed an NPS-mediated long-term (4 h after intranasal application) decrease in LTP and paired-pulse facilitation at CA3-CA1 synapses (Figure 1). The latter finding suggests that NPS activates presynaptic NPSRs at glutamatergic synapses in the VH, as it has previously been demonstrated to occur at synapses in the amygdala [7]. The observation of a decreased magnitude of LTP, however, rather argues for an additional postsynaptic localization of NPSRs on CA1 pyramidal neurons [41]. This scenario is also supported by the uptake of Cy3-NPS into these cells, which we detected both after its direct administration into the CA1 region (Figure 2) and after intranasal application [5].

Using two different analysis techniques (i.e. VSDI and fEPSP recording), we aimed to study different aspects of NPS-induced modifications of neuronal activity. Both the fast and long-lasting effects we observed point towards an NPS-mediated decrease in the overall activity and plasticity in the VH, which could in turn influence electrical activity within the amygdalar network, thereby contributing to the net anxiolytic effect of NPS.

To the best of our knowledge, this study is the first to relate effects of NPS to expression of functional NPSR in the hippocampus as shown by Cy3-NPS uptake and NPSR-specific antagonist actions (Figures 2A, 4B and 4C). Indeed, until now, NPSR expression in the hippocampus had only been described in the subiculum subfield in rodents [8], [42]. This discrepancy probably results from different sensitivities of the immunohistochemistry and *in situ* hybridization techniques used in these studies and the *in vivo* Cy3-NPS uptake approach employed here. While it is widely accepted that the PFC and the amygdala are key players in the brain's anxiety network [18], [43], [44], the role of the VH in anxiogenesis and anxiolysis is much less established. In humans, a nonmnemonic role for the hippocampus in emotional processing has hitherto not clearly emerged [45]. Recently, VH targeting has been shown to be sufficient for mediating the anxiogenic effect of CCK8 in rats [15]. Now, to our knowledge, we are the first to demonstrate that activity modulation in vCA1 is sufficient to mediate the effects of an exogenously applied *anxiolytic* agent, i.e. NPS. Taken together, these observations strongly suggest the VH as a potential target region for the pharmacotherapy of pathological anxiety. This hypothesis is in line with reports showing that lesions or inactivation of the VH result in a reduction of anxiety-like behavior in rodents [10], [46].

Interestingly, intra-vCA1-injected NPS did not significantly alter locomotion in any of the three behavioral tests employed (Figure 3B–D), while, in contrast, former studies reported ICV-injected NPS to induce hyperlocomotion [47], [48]. This discrepancy can be explained by the fact that local application targets only one specific brain structure, whereas ICV injections will influence several brain regions. Accordingly, intra-amygdalar injection of NPS also fails to elicit hyperlocomotion [7], [38].

Recent results from studies aiming at elucidating the projection sites of NPS-producing neurons show mismatches between NPSR expression and NPS fiber projections *inter alia* in the hippocampus [8], [42]. However, these findings do not interfere with our postulation of the VH as a player in NPS-elicited anxiolysis, since in our studies we concentrate exclusively on the targets and effects of *exogenously applied* NPS. These are of the utmost relevance for a potential future NPS-based anxiolytic therapy and must not necessarily overlap with targets and effects of endogenous NPS. Indeed, different effects of the endogenous NPS system and NPS treatment are most likely the case, as evidenced by the fact that, although strong anxiolytic actions of NPS treatment have been well established, mice lacking NPSR fail to display pathologically increased anxiety-like behavior [21].

In summary, our study provides the first experimental evidence for a direct involvement of the VH in NPS-induced anxiolysis, uncovers a novel mechanism of NPS action by showing NPS to rapidly weaken the spread of evoked electrical activity in the VH, and additionally demonstrates that intranasally applied NPS has the capacity to profoundly modulate glutamatergic synaptic transmission and plasticity in the VH. Since both the NPS/NPSR system [49] and the anatomy of the hippocampus [37] are highly conserved across mammals, it is likely that these findings can be translated to the situation in humans.

## Supporting Information

Figure S1 **Procedure of the intranasal application of NPS.** The awake mouse was restrained manually during the administration procedure in a supine position with the head immobile at an angle of approximatively 45° to the body.
(TIF)
