# Supplementary figures and images for: Identification of a Role for the Ventral Hippocampus in Neuropeptide S-Elicited Anxiolysis
Source: PLoS One. 2013 Mar 28;8(3):e60219. doi: 10.1371/journal.pone.0060219 (PMC3610821; doi:10.1371/journal.pone.0060219)

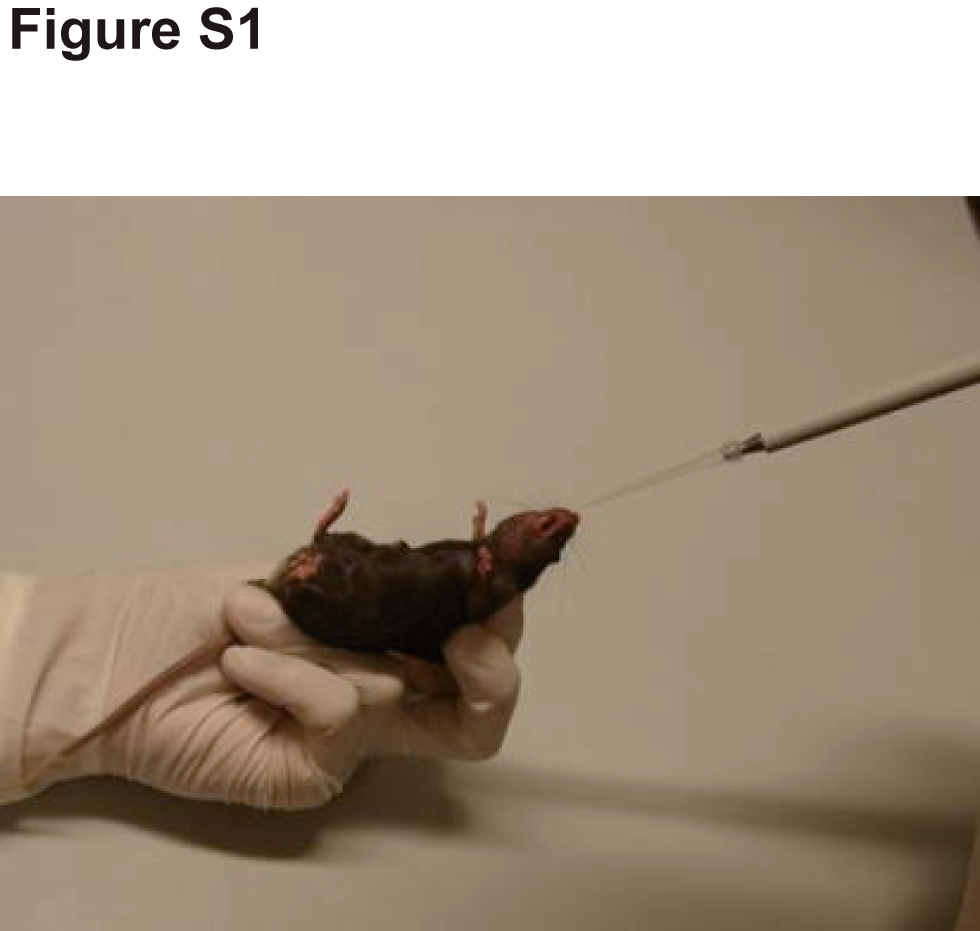

Supplement: Figure S1 — Procedure of the intranasal application of NPS. The awake mouse was restrained manually during the administration procedure in a supine position with the head immobile at an angle of approximatively 45° to the body. (TIF) [file pone.0060219.s001.tif]
